# High-performance lasers for fully integrated silicon nitride photonics

**DOI:** 10.1038/s41467-021-26804-9

**Published:** 2021-11-17

**Authors:** Chao Xiang, Joel Guo, Warren Jin, Lue Wu, Jonathan Peters, Weiqiang Xie, Lin Chang, Boqiang Shen, Heming Wang, Qi-Fan Yang, David Kinghorn, Mario Paniccia, Kerry J. Vahala, Paul A. Morton, John E. Bowers

**Affiliations:** 1 Department of Electrical and Computer Engineering, University of California, Santa Barbara, Santa Barbara, CA USA; 2 T. J. Watson Laboratory of Applied Physics, California Institute of Technology, Pasadena, CA USA; 3 Pro Precision Process and Reliability LLC, Carpinteria, CA USA; 4 Anello Photonics, Santa Clara, CA USA; 5 Morton Photonics, West Friendship, MD USA

**Keywords:** Semiconductor lasers, Silicon photonics

## Abstract

Silicon nitride (SiN) waveguides with ultra-low optical loss enable integrated photonic applications including low noise, narrow linewidth lasers, chip-scale nonlinear photonics, and microwave photonics. Lasers are key components to SiN photonic integrated circuits (PICs), but are difficult to fully integrate with low-index SiN waveguides due to their large mismatch with the high-index III-V gain materials. The recent demonstration of multilayer heterogeneous integration provides a practical solution and enabled the first-generation of lasers fully integrated with SiN waveguides. However, a laser with high device yield and high output power at telecommunication wavelengths, where photonics applications are clustered, is still missing, hindered by large mode transition loss, non-optimized cavity design, and a complicated fabrication process. Here, we report high-performance lasers on SiN with tens of milliwatts output power through the SiN waveguide and sub-kHz fundamental linewidth, addressing all the aforementioned issues. We also show Hertz-level fundamental linewidth lasers are achievable with the developed integration techniques. These lasers, together with high-*Q* SiN resonators, mark a milestone towards a fully integrated low-noise silicon nitride photonics platform. This laser should find potential applications in LIDAR, microwave photonics and coherent optical communications.

## Abstract (web summary)

Achieving high output power and low noise integrated lasers is a major challenge. Here the authors experimentally demonstrate integrated lasers from a Si/SiN heterogeneous platform that shows Hertz-level linewidth, paving the way toward fully integrating low-noise silicon nitride photonics in volume using real devices for lasing.

## Introduction

Silicon nitride photonics is emerging in recent years as advanced photonic devices require better performance than is available from traditional Si or InGaAsP-based waveguides^1^. As a fully CMOS-compatible material, SiN-based waveguides offer low optical propagation loss, wide wavelength transparency from the visible to the infrared, a low thermo-optic coefficient, and absence of nonlinear absorption loss^2–5^, thus forming the backbone of chip-scale nonlinear photonics^6–9^, high-fidelity integrated microwave photonics systems^10^ and ultra-broadband integrated photonic circuits^11,12^. As a result, SiN-based photonic components, benefiting from the superior passive properties of SiN material, represent the state-of-the-art performance of integrated photonics, including frequency comb generators^13–16^, optical gyroscopes^17^, radio-frequency filtering^18^ and so on.

However, SiN photonics has been largely restricted at the stand-alone component level as the integration with active devices including lasers, modulators, amplifiers, and photodetectors has been difficult. First of all, as a dielectric material, SiN lacks a direct energy bandgap for efficient carrier radiative recombination, or electro-optic effect, which are the basis of lasers and modulators respectively. In addition, the refractive index of SiN_*x*_ at telecommunication wavelength (1.55 µm) is around 2, depending on the silicon content. This low refractive index possesses significant difficulties in its integration with active III–V gain materials through direct heterogeneous III-V/SiN integration, analogous to heterogeneous III–V/Si integration, which has achieved success in optical interconnect applications^19–21^.

Heterogeneous integration with III–V materials is still the most viable approach for fully integrated lasers on SiN^22–24^. Recent progress in multilayer integration leverages an intermediate Si layer as the index matching layer to create III-V/Si/SiN structures^22^. This structure not only provides optical gain to SiN photonic circuits so that lasers or amplifiers can be formed, but also enriches the photonic functionalities of SiN photonic circuits as optical modulation^25^ and detection^26^ are enabled using existing III–V/Si or Si devices. Another compelling opportunity through the integration of ultra-low-loss SiN into various photonic devices is that the device performance can be optimized with another degree of freedom. One prominent example is the semiconductor laser linewidth, which is largely restricted to the MHz range for monolithic III–V lasers^27^. The underlying prospect is that low-loss Si waveguides and ultra-low-loss SiN waveguides can offer orders-of-magnitude longer optical cavities compared to III–V-based waveguides, thus reducing the laser linewidth. Sub-kHz and Hz-level fundamental linewidth semiconductor lasers are demonstrated through heterogeneous or hybrid integration of III-V gain with low-loss Si^28–30^ or SiN^31–34^. As SiN waveguides offer orders of magnitude lower loss than state-of-the-art Si waveguides, it is ideal to build a fully integrated low-noise photonics platform based on SiN. High-power, low-noise semiconductor lasers are of critical importance in many applications including coherent communications, LIDAR and remote sensing^35^. Fully integrated lasers on SiN eliminate the need for free space or optical fiber coupling to SiN waveguides, improving both the device scalability and stability.

Here, we demonstrate high-power (>10 mW), low-noise (<1 kHz fundamental linewidth) and <−150 dBc/Hz RIN (relative intensity noise) lasers using multilayer heterogeneous integration. These lasers, in a proven integration scheme with ultra- high-*Q* SiN microresonators, yield Hertz-level fundamental linewidth on-chip lasers and can also directly generate optical frequency combs. Our high-performance lasers could be a key enabler for fully integrated SiN photonics, featured with on-chip frequency comb generation capability^36–38^ and ultra-low phase noise^33^, to be used in high-fidelity microwave photonics^39–42^, high channel-count DWDM (dense wavelength division multiplexing) systems^43^ and so on.

## Results

### Laser design

**Fig. 1:**
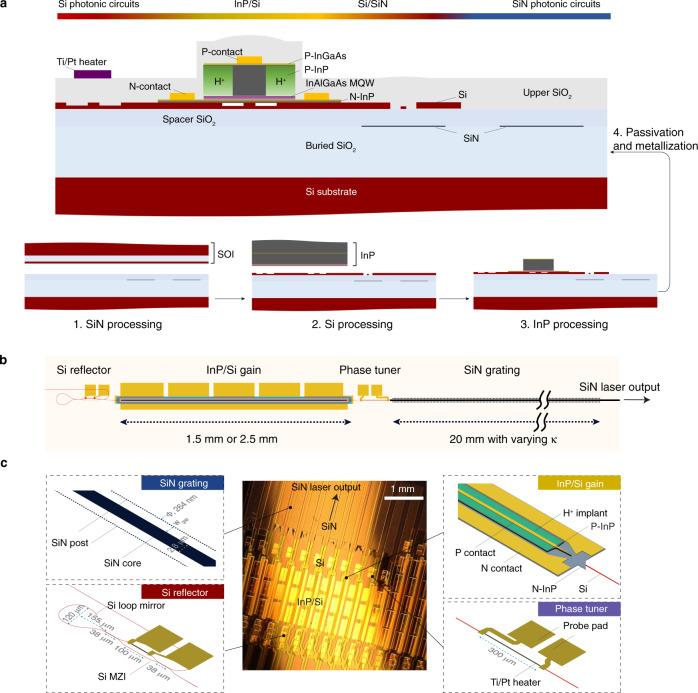
Laser design and fabrication schematics. **a** A cross-sectional schematic illustration of the fabricated InP/Si/SiN laser integrated with Si photonic circuits and SiN photonic circuits after the laser passivation and before probe metal deposition (top). Three insets on the bottom show the fabrication process including SiN processing and SOI bonding (left), Si processing and InP bonding (middle), and InP processing (right). MQW multiple quantum well. **b** Top-view schematic illustration of the E-DBR laser. **c** A device optical photograph showing InP/Si/SiN DBR laser arrays. The lasers consist of four sections including the SiN grating, InP/Si gain section, Si reflector, and phase tuner, with schematics and dimensions shown, respectively.

The laser is constructed in a way that the Si layer, sandwiched between the III–V epi layer and SiN layer, bridges the refractive indexes and provides additional in-cavity phase control capability. As shown in Fig. 1a, two wafer bonding steps, including SOI bonding and InP bonding, follow SiN processing and Si processing, respectively. SOI bonding uses a large piece of SOI (500-nm-thick Si layer) to cover the entire device area including laser gain area and SiN photonic circuits, which helps to maintain the low loss of SiN waveguides during III–V processing. As a result, in addition to standard III-V processing as in III-V/Si lasers, the removal of excess Si on top of the SiN photonic circuits except the taper needs to be performed before laser passivation. The details of fabrication can be found in “Methods”.

Equipped by this multilayer structure, our laser contains the following main sections as shown in the laser schematics (Fig. 1b) and details in Fig. 1c: SiN Bragg grating, InP/Si gain, Si reflector, and phase tuner. The SiN grating is a low-*κ* (coupling constant) side post grating, made by placing SiN posts along the SiN waveguide core on both sides. The gap width (*W*_gap_) between the core and posts is constant and can be tailored to achieve the desired *κ* value. In order to achieve a long cavity length with high single-mode selectivity, the gratings use the maximum available 20 mm in length to fit the laser within a deep ultra-violet (DUV) stepper mask reticle, providing an extended-distributed Bragg reflector (E-DBR). The grating length, however, can be further extended if using a spiral-shaped grating structure^22^. The InP/Si gain section uses a hybrid InP/Si active waveguide with mode transition tapers to the underneath Si waveguide. The taper transition loss at this stage is below 1 dB and the details of this type of hybrid section can be found in the previous work^44^. The gain section lengths are chosen to be 2.5 mm or 1.5 mm in different laser designs. Here the Si waveguide is shallow etched with 231-nm etch depth to support single transverse-electric (TE) mode in the hybrid InP/Si waveguide section. After the formation of the wide InP mesa and laser passivation, proton implantation is implemented to define the electrical current channels for efficient carrier injection. While the SiN grating provides narrow-band feedback for one mirror, the other mirror is a broadband Si reflector based on a tunable Si loop mirror. A thermally controlled Si MZI (Mach–Zehnder interferometer) is used to tune the interferometer power splitting ratio and consequently, the total reflectivity. The Si reflector is designed to provide 100 % power reflectivity with zero bias as the initial state. The dimensions of the Si loop mirror are shown in Fig. 1c. A phase tuner using a thermally tuned heater (Ti/Pt, 300-µm long) is included between the InP/Si gain and SiN grating, to tune the intra-cavity phase condition. The E-DBR laser output is taken through the SiN grating in a SiN waveguide, followed by a SiN inverse taper for efficient chip-to-fiber coupling and off-chip laser characterization. The laser output could also be directed to other SiN photonic integrated components on demand.

In our E-DBR lasers, the SiN waveguide is 90-nm thick and 2.8-µm wide, with an effective mode refractive index around 1.46 and an effective mode area of 5.3 µm^2^. In order to enable a highly efficient mode conversion between the Si waveguide and SiN waveguide, the shallow-etched Si waveguide mode is transformed to 269-nm-thick Si fully etched waveguide mode and then tapered to a 150-nm-wide taper tip over a 50-µm length. The simulated taper transmission loss is below 0.5 dB. Cut-back test structures with a series of tapers could be included to experimentally extract the taper loss. The laser fabrication is a wafer-scale process, which is performed on a 100-mm diameter wafer and all the optical lithography is performed using a DUV stepper. The device scale is determined upon the device layout plan and the current wafer demonstrated 60 mm × 60 mm SOI to SiN bonding. This scale can be extended to full 100-mm wafer scale to enable more devices using the same process.

### Laser characterization

**Fig. 2:**
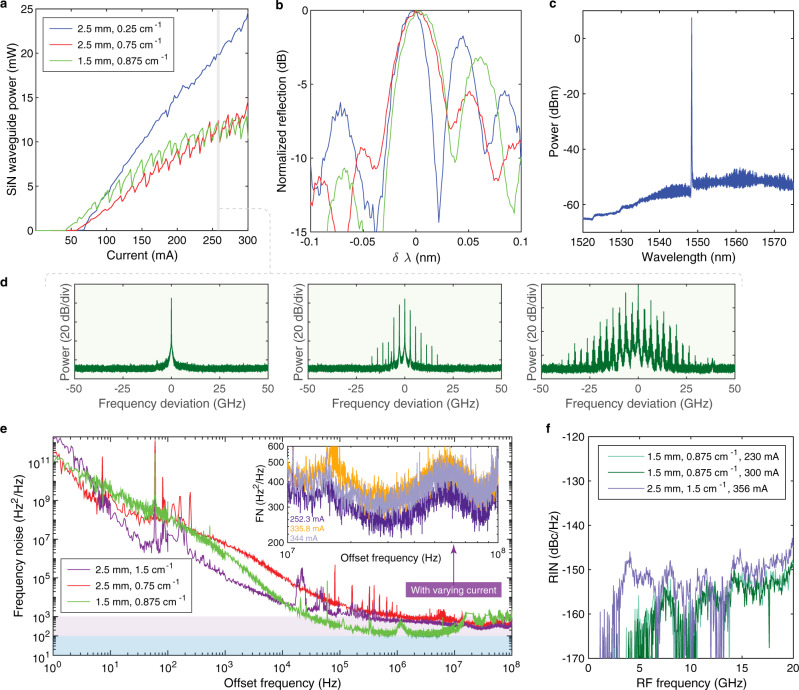
Laser characterization. **a** LI measurements of three lasers with different gain section length (1.5 mm or 2.5 mm) and grating *κ* values (0.25 cm^−1^, 0.75 cm^−1^, and 0.875 cm^−1^). **b** Corresponding grating reflection response of lasers measured in (**a**). **c** Optical spectrum of the high-power laser (shown in blue in **a** and **b**) at a gain current of 300 mA. The optical spectrum analyzer resolution bandwidth is 0.02 nm. **d** Single-mode and multimode regimes as the wavelength are red-tuned across a mode-hop cycle, commonly observed in DBR lasers^29, 35^. The corresponding laser gain currents from left to right are 254.4, 258, and 261 mA, respectively. **e** Laser frequency noise spectrum of lasers with low-frequency noise and either kHz or sub-kHz level fundamental linewidth. The laser currents are 252.3 mA (purple), 382.2 mA (red) and 298 mA (green). Inset shows current-dependent high-offset frequency noise spectra for the laser with 2.5 mm gain section length and 1.5 cm^−1^ grating *κ* value (purple), indicating sub-kHz level Lorentzian linewidth is achieved for all three currents. **f** Laser RIN measurements of a low-threshold laser (shown in green in **a** and **b**) and another laser (shown in purple in **e**).

Figure 2 summarizes the laser characterization results. The laser output power is measured from the output-coupled optical lensed fiber. The coupling loss is 3.5 dB from the SiN inverse taper at the chip facet. The CW (continuous-wave) light-current (LI) curves are shown in Fig. 2a for three lasers working at 20 °C stage temperature with different gain section lengths (1.5 mm or 2.5 mm) and different SiN grating *κ* designs (0.25 cm^−1^, 0.75 cm^−1^, and 0.875 cm^−1^). The circular grating post is 263 nm in diameter and the waveguide-post gap widths *W*_*gap*_ of gratings used in these lasers are 1.783, 1.338, and 1.276 µm. The SiN output waveguide power is over 10 mW for each of these lasers. The LI measurements show that a long gain section length together with a small grating *κ* value (weak grating feedback) are advantageous for high laser output power, as a small *κ* corresponds to a larger mirror loss at the output facet. The minimum *κ* value, which represents the minimum grating feedback strength sustaining lasing conditions, is determined with a combination of factors including the laser gain, intrinsic loss, Si/SiN taper loss, and SiN waveguide loss^31^. The laser threshold also depends on the laser gain section length and the grating feedback. A short gain section length of 1.5 mm together with a large grating *κ* value of 0.875 cm^−1^ results in the low laser threshold of 42 mA for the long external cavity laser (20-mm-long external cavity). The extracted threshold current density for each of these lasers shown in Fig. 2a is 580 A/cm^2^ (red), 680 A/cm^2^ (blue), and 700 A/cm^2^ (green). As the lasers have a thick underneath buried oxide layer, the laser performance (i.e. output power) could be further improved by using thermal shunt designs to dissipate heat through the Si substrate^45,46^. However, this requires additional processing steps and the current lasers did not show significant thermal roll off at elevated gain currents.

The SiN grating reflection responses for the three lasers in Fig. 2a are shown in Fig. 2b. From the normalized reflection, it is clear that the small grating *κ* design results in a narrower reflection bandwidth, however, with limited side lobe suppression (3.5 dB). For larger *κ* values, the grating reflection bandwidth increases. The side lobe suppression has a maximum ratio at certain *κ* and sees a decrease with further increased *κ* value, as indicated by the red and green curve in Fig. 2b. The side lobes of the grating spectra are not fully symmetric, which are likely due to the slight grating post size variation (on the order of nanometers) along the long grating length (20 mm). However, the gratings already provide enough single-mode selectivity together with narrow bandwidth for sub-kHz Lorentzian linewidth lasing, while further optimized grating uniformity could potentially push the laser linewidth down to sub-100 Hz level.

As the SiN grating provides the narrow-band reflection and the Si reflector feedback is broadband, the laser wavelength is determined by the SiN grating. Here the grating period is 526 nm and the lasing wavelength is around 1548 nm. Single-mode operation is achieved with proper phase conditions and the single-mode laser outputs high power within the mode-hop cycles as shown in Fig. 2a. Side mode suppression ratio as high as 54 dB is achieved for the high-power laser with 2.5-mm-long gain section and 0.25 cm^−1^ grating *κ*, at a gain current of 300 mA (Fig. 2c).

The kinks shown in the LI curves in Fig. 2a are due to the laser mode hops between different longitudinal mode states. These mode-hops are determined by the intra-cavity optical phase^22^. Single longitudinal mode states are preferred and discussed throughout this paper. At certain gain currents, the lasers work in multimode states, when gain competition results in multiple longitudinal modes lasing. These states are shown as examples in Fig. 2d, which correspond to the laser with 1.5 mm gain length and 0.875 cm^−1^ grating *κ* operating at 254.4 mA, 258 mA and 261 mA, within a mode-hop cycle. The stronger gratings result in more mode hops as the stronger grating provides a wider grating reflection bandwidth, which would favor multiple longitudinal modes for multimode lasing.

The extended SiN grating provides a long external cavity which enables a narrow laser linewidth. For an external cavity laser, the fundamental linewidth ∆*υ* = ∆*υ*_0_*/*(1 + *A* + *B*)^2^, where ∆*υ*_0_ is the laser Schawlow-Townes linewidth without external feedback (determined by the III-V/Si gain section), *A* is the ratio of external passive Si and SiN cavity length to the active III- V/Si gain section length, and *B* includes the detuned loading effect which requires slight red-detuning of the lasing wavelength relative to the grating reflection peak^47–49^. The frequency noise spectra of three lasers with different gain section lengths and *κ* values are shown in Fig. 2e. For each of the lasers, due to the splits on the grating *κ* and gain section length, the frequency noise performance varies. However, all the lasers provide kHz or sub-kHz level Lorentzian linewidth: 1 kHz (red), 780 Hz (purple), and 400 Hz (green). The narrow Lorentzian linewidth results from the combined effect of extended long grating cavity and detuned loading. The inset of Fig. 2e shows high-offset frequency noise of the laser with 2.5-mm long gain section length and cm^−1^ grating *κ* at varying currents locating at three different single mode operation regimes. All the frequency noise traces exhibit sub-kHz Lorentzian linewidth. The laser single-mode operation is stable, and the frequency noise measurement results are verified repeatedly with same laser gain current settings. The measurement results shown are taken when the laser chip is placed on a temperature-controlled stage with 20 °C stage temperature. The lasers can lase at up to 75 °C stage temperature, with low wavelength variation due to the low thermo-optic coefficient of SiN and SiO_2_^50^. We also measured the RIN of the 1.5-mm long gain section laser (0.875 cm^−1^ grating *κ*). The results are shown in Fig. 2f. For gain currents around 230 mA and 300 mA, the RIN are measured below −150 dBc/Hz up to 20 GHz. This low RIN is due to the narrow bandwidth feedback from the SiN grating. Another laser, with a 2.5-mm-long gain section laser and 1.5 cm^−1^ grating *κ*, shows slightly higher RIN due to a longer gain section and wider grating response, but still below −148 dBc/Hz.

### Integration with ultra-high-*Q* SiN photonics

With the laser fully integrated with SiN photonic integrated circuits, its performance can be further enhanced by the addition of ultra-low-loss and ultra-high-*Q* SiN photonic devices. Moving towards an ultra-low phase noise system, our lasers can be integrated with ultra-high-*Q* SiN ring resonators. As a proof-of-concept, here we place our SiN laser chip in close proximity with a stand-alone SiN high-*Q* ring resonator chip for butt coupling.

**Fig. 3:**
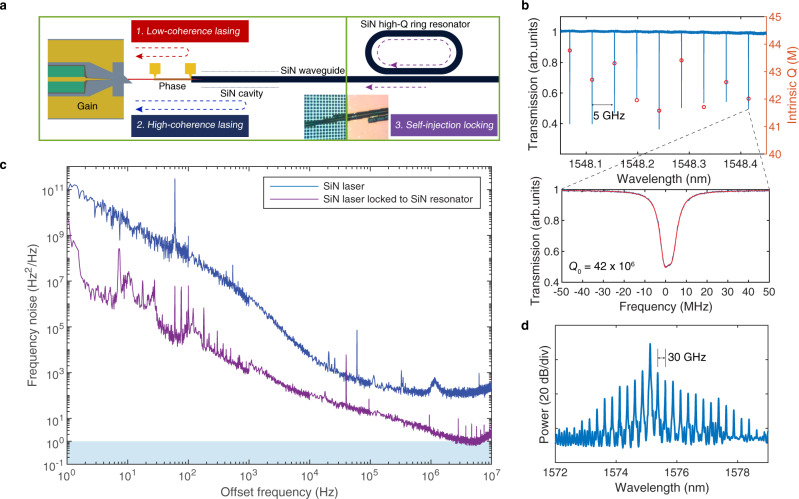
Heterogeneous SiN lasers self-injection locking to high-*Q* SiN ring resonators. **a** Schematic illustration of three potential lasing conditions with a III–V/Si/SiN platform. Each condition relies on different feedback mechanism and results in different laser coherence. **b** Transmission of a high-*Q* SiN ring resonator with 5-GHz FSR and the corresponding intrinsic *Q* factors around the SiN laser resonance. Bottom shows the zoom-in resonance transmission used for self-injection locking with fitted curve (shown in red). **c** Laser frequency noise of a high-coherence SiN laser at free-running state (blue) and self-injection-locked state (purple) to a high-*Q* SiN ring resonator. **d** Optical spectrum showing frequency comb generation of a high-power laser on III-V/Si/SiN platform self-injection locked to a 30-GHz FSR high-*Q* ring resonator with normal dispersion.

Figure 3a summarizes the influence of external cavity feedback from the resonator on the laser coherence. Within the E-DBR laser, due to the large mode mismatch between the Si and SiN waveguides, efficient mode transition with low reflection is only possible when the taper tip is narrow. On the other hand, in devices where the taper tip is wider and provides enough feedback, the laser can lase from the wide taper tip and high-reflection reflector (on the other side) in a Fabry–Pérot (FP) scheme (in mode competition with SiN grating-based lasing), with the output coupled to the SiN waveguide. This results in a low-coherence lasing condition, lasing from a coupled FP cavity formed by the series of tapers (III–V/Si to Si taper and Si to SiN taper) and the Si reflector. Due to the lack of long external cavity, the laser exhibits very low coherence, i.e. a broad laser linewidth. These lasers, with absences of the SiN cavity loss and Si–SiN transition loss in the laser cavity, could result in lasers that are advantageous for applications that require higher output power or lower laser coherence, e.g., as frequency-modulated lasers for optical gyroscopes^51^.

For a properly designed and highly efficient Si/SiN transition taper with low back-reflection, the laser feedback is provided by the SiN grating, enabling high-coherence lasing and (sub-) kHz fundamental linewidth. To achieve this, the Si waveguide taper tip needs to be made narrow to achieve high transmission efficiency to the underneath SiN waveguide and avoid low-coherence lasing. In this case, the SiN grating is part of the external cavity and needs to be low-loss to provide enough feedback to facilitate SiN grating-based lasing. However, as the grating length is limited by its physical length (the grating effective cavity length is always shorter than physical length), this approach cannot be extended beyond the physical cavity length limit. Alternatively, resonant structures like ring resonators offer optical length enhancement depending on *Q* factor and the bus waveguide to ring resonator coupling ratio.

To further reduce the laser linewidth, one solution is to self-injection lock the laser with an ultra-high-*Q* ring resonator on the same SiN platform. Both E-DBR and FP lasers work well with external resonator feedback in self-injection locked operation; the E-DBR providing the best frequency noise results (Fig. 3c), while the FP-based operation can be used to generate frequency combs once it exceeds the parametric oscillation threshold (Fig. 3d). Our E-DBR laser and the SiN resonator use identical facet inverse taper and identical SiN waveguide geometry. The adopted SiN ring resonator has 5-GHz free spectral range (FSR). The measured *Q* factor around the laser wavelength (1548 nm) is shown in Fig. 3b. For the resonance used for the laser self-injection locking, the intrinsic *Q* factor is around 42 million. Laser self-injection locking happens when the laser wavelength coincides with the SiN ring resonance, where the feedback signal results from Rayleigh scattering at the high-*Q* resonance within the ring resonator. The E-DBR laser used here has 1.5-mm gain section length and 0.875 cm^−1^ grating *κ* (shown as Fig. 2a, e  green), and its frequency noise before and after self-injection locking is shown in Fig. 3c. It can be seen that the laser frequency noise is reduced by around 20–30 dB across the full frequency range, resulting in below 1 Hz^2^/Hz white noise floor, corresponding to about 3 Hz Lorentzian linewidth. The phase noise can be further decreased by introducing an add-drop ring configuration and taking the laser output from the drop port of the ring resonator to use the ring resonator as a low-pass filter (by a few times), or using a SiN ring resonator with larger mode volume to reduce the thermorefractive noise (by another one or two orders depending on the design)^33^. The coupling loss between these two chips is measured to be 3 dB since the SiN high-*Q* resonator chip facet is not polished. As a result, the current hybrid-integrated ensemble without resonance locking has half the output power compared with the free-running stand-alone laser chip. With fine polishing or heterogeneous integration, the coupling loss is negligible since the waveguide cores have identical dimensions, which could enable better device performance. The current integration platform can also be leveraged to enable widely tunable heterogeneously integrated lasers with Hertz-level linewidth if the laser is replaced by widely tunable III-V/Si lasers (based on Si ring resonators for tuning), heterogeneously integrated with low-FSR high-*Q* SiN ring resonators for self-injection locking across a wide wavelength range.

Another capability with this integration platform is optical frequency comb generation, as the laser output power exceeds the parametric oscillation threshold of high-*Q* SiN ring. Figure 3d shows frequency comb generation from a high-*Q* SiN ring resonator with 30-GHz line spacing, directly pumped by a low-coherence FP laser from a SiN waveguide output. No other comb states are achieved in the current lasers since the laser output power (especially with the present 3 dB chip-to-chip coupling loss) is insufficient for pulse generation. With further increased output power, dark pulses are achievable using this integration platform with normal-dispersion SiN microresonators^33^. The direct pumping scheme using laser self-injection locking can also be extended using our platform with dispersion-engineered thick SiN microresonators for bright soliton generation. High Si bonding yield was achieved on a thick SiN wafer with proper chemical–mechanical polishing (CMP) and the current integration approach has been experimentally verified^16^. This fully integrated III-V/Si/SiN structure can thus form an ultra-low-noise laser and nonlinear photonics platform for various applications including coherent optical communications^52^, optical clocks^53^ and optical/RF frequency synthesizers^54^.

## Discussion

In summary, we have demonstrated high-power, low-noise lasers heterogeneously integrated with SiN photonic integrated circuits. The laser also enables Hertz-level instantaneous linewidth laser output and optical frequency comb generation on the same platform, paving the way for next-generation ultra-low-noise integrated photonics for a wide variety of applications. Moreover, our integration platform can be extended to other material systems by replacing either the gain medium (e.g. quantum dots^55^) or low-loss waveguide material (e.g., lithium niobate^56–58^), enabling seamless integration of high-performance lasers in various photonic platforms.

## Methods

### Device fabrication

Laser fabrication starts from a thermally grown SiO_2_ (8-µm thick) on Si wafer with 100-mm diameter. Ninety-nanometer-thick stoichiometric Si_3_N_4_ is deposited by low-pressure chemical vapor deposition. SiN waveguide patterning together with SiN grating fabrication are performed using 248-nm DUV lithography followed by inductively coupled plasma (ICP) etching using CHF_3_/CF_4_/O_2_ gas. Deuterated SiO_2_ of 900 nm is deposited on top of the SiN waveguides forming the first layer of the waveguide cladding. This cladding layer is planarized by CMP and the resultant cladding thickness is around 600 nm. A large silicon on insulator (SOI) piece (60 mm × 60 mm) is bonded on the wafer covering device areas using oxygen-assisted plasma-activated bonding. The bonded SOI size can be extended to a full 100-mm-diameter wafer, if additional treatments can be done to allow enough clearance area for alignment markers which are written in the SiN layer. Si substrate removal is done by a combination of mechanical lapping and Si Bosch etching. The buried oxide layer of bonded SOI piece is removed with buffered hydrofluoric acid. Si waveguide patterning is done by DUV stepper and C_4_F_8_/SF_6_ based reactive-ion etching (RIE). After the Si waveguide formation, InP MQW epi is bonded on the laser gain area, followed by InP substrate mechanical lapping and HCl acid etching. The substrate acid wet etching stops at the p-InGaAs layer. The InP laser mesa is formed by CH_4_/H_2_/Ar based dry etching and H_3_PO_4_ based wet etch for the MQW region. Excess Si on top of SiN photonic circuits is removed by XeF_2_ isotropic gas etch. The laser is then passivated by in-total 900-nm thick deuterated SiO_2_, which also forms the second layer of SiN waveguide cladding. Vias are then opened, and N-contact metal (Pd/Ge/Pd/Au) and P-contact metal (Pd/Ti/Pd/Au) are deposited using electron-beam deposition and a lift-off process. Proton implantation follows the contact metal formation. Heater and probe metal are then deposited. The laser chips are diced and polished so that the SiN inverse taper facet is exposed for laser characterization.

### Laser self-injection locking

The InP/Si/SiN laser is mounted on a temperature-controlled ceramic stage, and the probe pads are wire bonded in a butterfly-type package. The whole package can be positioned on an XYZ and rotation stage for butt coupling to a SiN high-*Q* ring resonator chip which is mounted on a separate stage. The laser injection current is tuned to match the laser wavelength to a ring resonance. Self-injection locking is confirmed from the output power on a photodiode (Thorlabs DET01CFC, a large dip in the output power when the locking state is achieved), together with a fiber unbalanced-MZI based wavemeter for monitoring laser frequency stability, where a locked state shows quiet output power trace in the time-domain after the wavemeter on a real-time oscilloscope (Tektronix MSO64).

### Laser noise measurement

The laser frequency noise is measured using an OEWaves OE4000 laser linewidth/phase noise measurement system. The laser RIN measurement is performed by applying the fiber-coupled light into a high-speed photodetector (Discovery DSC30) followed by a low-noise amplifier and microwave spectrum analyzer (Agilent E4448A). The noise measurement was taken from 1 to 20 GHz, from which the photodetector and amplifier frequency response are subtracted. Thermal noise and shot noise (calculated from the photodetector current) are also removed. The laser RIN traces in Fig. 2f show very low values below 5 GHz (≤−165 dBc/Hz), with some peaking up to −155 dBc/Hz at 8 and 12 GHz caused by interactions between the lasing mode and close-in cavity modes, while above 14 GHz the measurements follow the noise floor of the spectrum analyzer.

### Reporting summary

Further information on experimental design is available in the Nature Research Reporting Summary linked to this paper.

## Supplementary information

Lasing Reporting Summary

Authors Checklist

## Data Availability

The data that support the plots within this manuscript and other findings of this study are available on Zenodo (10.5281/zenodo.5565401).
