# Supplementary material for: High-performance lasers for fully integrated silicon nitride photonics
Source: Nat Commun. 2021 Nov 17;12:6650. doi: 10.1038/s41467-021-26804-9 (PMC8599668; doi:10.1038/s41467-021-26804-9)
Supplement: Supplementary file 1 — Lasing Reporting Summary [file 41467_2021_26804_MOESM1_ESM.pdf]

nature research

Corresponding author(s): John E. Bowers

Lasing Reporting Summary

Nature Research wishes to improve the reproducibility of the work that we publish. This form is intended for publication with all accepted papers reporting claims of lasing and provides structure for consistency and transparency in reporting. Some list items might not apply to an individual manuscript, but all fields must be completed for clarity.

For further information on Nature Research policies, including our data availability policy, see Authors & Referees.

Experimental design

Please check: are the following details reported in the manuscript?

1. Threshold

Plots of device output power versus pump power over a wide range of values indicating a clear threshold

[x] Yes
[ ] No

The information on laser threshold is clearly presented in Fig. 2a and related text in the section: 'Laser characterization'.

2. Linewidth narrowing

Plots of spectral power density for the emission at pump powers below, around, and above the lasing threshold, indicating a clear linewidth narrowing at threshold

[ ] Yes
[x] No

The optical spectrum (Fig. 2c) above threshold with high side mode suppression is a clear indication of lasing. No below or around lasing threshold data is necessary.

Resolution of the spectrometer used to make spectral measurements

[x] Yes
[ ] No

We specified "The optical spectrum analyzer resolution bandwidth is 0.02 nm" in Fig. 2c caption.

3. Coherent emission

Measurements of the coherence and/or polarization of the emission

[x] Yes
[ ] No

The laser coherence (phase noise) is measured and presented in Fig. 2e and discussed in related text in the section: 'Laser characterization'.

4. Beam spatial profile

Image and/or measurement of the spatial shape and profile of the emission, showing a well-defined beam above threshold

[ ] Yes
[x] No

We use fiber coupled power for laser characterization and the edge emitting laser beam is well-defined.

5. Operating conditions

Description of the laser and pumping conditions
Continuous-wave, pulsed, temperature of operation

[x] Yes
[ ] No

In the section :'Laser characterization', we specify "The CW (continuous-wave) light-current (LI) curves are shown in Fig. 2a for three lasers working at 20 oC stage temperature ..."

Threshold values provided as density values (e.g. W cm^{-2} or J cm^{-2}) taking into account the area of the device

[x] Yes
[ ] No

In the section :'Laser characterization', we specify "The extracted threshold current density for each of these lasers shown in Fig. 2a are 580 A/cm2 (red), 680 A/cm2 (blue) and 700 A/cm2 (green)."

6. Alternative explanations

Reasoning as to why alternative explanations have been ruled out as responsible for the emission characteristics
e.g. amplified spontaneous, directional scattering; modification of fluorescence spectrum by the cavity

[ ] Yes
[x] No

Our LI data in Fig. 2a and spectrum data in Fig. 2c are clear evidences of electrically-pumped semiconductor lasers.

7. Theoretical analysis

Theoretical analysis that ensures that the experimental values measured are realistic and reasonable
e.g. laser threshold, linewidth, cavity gain-loss, efficiency

[ ] Yes
[x] No

The experimental values are reasonable and are collected using calibrated laser measurement equipments. The theoretical analysis on laser threshold etc. is not relevant with this paper.

8. Statistics

Number of devices fabricated and tested

[ ] Yes
[x] No

Statistics summary of number of devices are not relevant with this paper.

Statistical analysis of the device performance and lifetime (time to failure)

[x] Yes
[ ] No

We have presented device performances for lasers with different designs and at different operation conditions (Fig. 2e and Fig. 2f.). No lifetime data is presented since it is not relevant with this paper.
