# Supplementary material for: High-performance lasers for fully integrated silicon nitride photonics
Source: Nat Commun. 2021 Nov 17;12:6650. doi: 10.1038/s41467-021-26804-9 (PMC8599668; doi:10.1038/s41467-021-26804-9)
Supplement: Supplementary file 2 — Authors Checklist [file 41467_2021_26804_MOESM2_ESM.docx]

| 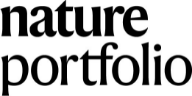 |  |
| --- | --- |
| **Author Checklist** | NCOMMS-21-13031A-Z |
| **0000000000000000000000000000000000000000000000000000000000000** | **0000000000000000000000000000000000000** |
| **Please check the items below carefully and add a response in each row of the table to indicate the changes that you have made. Please also check through any additional marked-up edits we have may have provided within the manuscript file.** | |
|  |  |
|  |  |
|  |  |
| Abstract and editor's summary |  |
| Our guidance: | Your response: |
| Your paper will be accompanied by the following editor's summary. Please let us know if there are any inaccuracies: 'Achieving high output power and low noise integrated lasers is nowadays a challenge. Here the authors experimentally demonstrate lasing form a Si/SiN heterogeneous platform that show Hertz-level linewidth paving the way toward the possibility to fully integrate low-noise silicon nitride photonics in real devices for lasing.' | What your wrote is acceptable. I think this is a little better: 'Achieving high output power and low noise integrated lasers is a major challenge. Here the authors experimentally demonstrate integrated lasers from a Si/SiN heterogeneous platform that shows Hertz-level linewidth, paving the way toward fully integrating low-noise silicon nitride photonics in volume using real devices for lasing.' |
|  |  |
| Author information |  |
| Our guidance: | Your response: |
|  |  |
| Please review your complete author list to verify that it is complete and accurate. We ask that you consult with your coauthors to ensure that all names, affiliations, and titles are represented correctly. Note that if any authors are added or removed after this point then all authors will be requested to provide approval documentation that could potentially delay the production of your paper. | We have reviewed the author list and confirmed its correctness. |
|  |  |
| Article structure |  |
| Our guidance: | Your response: |
| We can accommodate up to 10 display items (Figures or Tables) in the main article. Each Figure and Table must fit easily within an A4 page (210 x 297 mm). Please ensure that the number and size of your Figures and Tables fulfil these requirements to avoid any delay in the acceptance of your article. | We have 3 Figures in the main article and they all fit easily within an A4 page. |
| **Please ensure your main manuscript file includes the following sections, in this order:** | We have confirmed our paper is organized as follows: |
| *Title Author list Affiliations Abstract Introduction Results Discussion (optional) Results and Discussion (optional) Methods (including Data Availability, Code Availability and Statistics subsections where relevant) References Acknowledgements Author Contributions Statement Competing Interests Statement Tables Figure Legends/Captions (for main text figures)* | *Title Author list Affiliations Abstract Introduction Results Discussion  Methods  References Acknowledgements Author Contributions Statement Competing Interests Statement Figure Captions (for main text figures)* |
| We do not edit Supplementary Information files; they will be uploaded with the published article as they are submitted with the final version of your manuscript. Any tracked changes should be removed from the file and the file should be provided as a PDF file. Supplementary Figures do not need to be provided separately. |  |
|  |  |
|  |  |
| Main text |  |
| Our guidance: | Your response: |
| Please rearrange the Introduction so that all discussion of previous work appears first. The final paragraph should contain only a concise summary of the current work, in the present tense, and begin with a phrase like “In this work” or “Here, we show”. | We confirmed our paper follows the guide. |
| Please do not use italics, bold font, underlining or speech marks unless required for technical terms (in both the main text and the display items). | We confirmed our paper follows the guide. |
| Please make sure that mathematical terms throughout your manuscript and Supplementary Information (including in figures, figure axes, and legends) conform strictly to the following guidelines. Equations must be supplied in editable format, and not as images. Scalar variables (e.g. x, V, χ) must be typeset in italic, whereas multi-letter variables and functions (e.g. log) must be formatted in roman. Vectors (such as the wavevector k or the magnetic field vector B) must be typeset in bold without italics. | We confirmed our paper follows the guide. |
| Please divide the Results section into subsections, each with a title of 60 characters or fewer including spaces. | We confirmed our paper follows the guide. |
|  |  |
| Figures and Tables |  |
| Our guidance: | Your response: |
| Please see the guidelines linked below for detailed instructions about how your figures should be prepared. Following these instructions will reduce the chances of delays should we need to request replacement artwork from you at a later stage. | We confirmed our paper follows the guide. |
| <https://www.nature.com/documents/NRJs-guide-to-preparing-final-artwork.pdf> |  |
| Shadings or symbols in graphs must be defined in some fashion. We prefer that you use a key within the image; do not include colored symbols in the legend/caption. | We confirmed our paper follows the guide. |
| Please make sure that the terms ‘atomic units (a. u.)’ or ‘arbitrary units (arb. units)’ are appropriately used. | We confirmed our paper follows the guide. |
| Any abbreviations, symbols or colours present in your figures must be defined in the associated legends. | We confirmed our paper follows the guide. |
|  |  |
| Data and Code |  |
| Our guidance: | Your response: |
| Nature journals strongly support public availability of data and code. Please deposit the data and code used in your paper into a public data repository, or alternatively, present the data as Supplementary Information. If data can only be shared on request, please explain why in your Data Availability Statement, and also in the correspondence with your editor.   Please note that for some data types, deposition in a public repository is mandatory. Any restrictions on sharing of these data types must be clearly indicated in the statement and discussed with the editor. More information on our data deposition policies and available repositories can be found here: | We uploaded all the data that generated the figures in our paper to Zenodo and this information is included in the ‘Data Availability’ statement:  The data that support the plots within this manuscript and other findings of this study are available on Zenodo (https://doi.org/10.5281/zenodo.5565401). |
| <https://www.nature.com/nature-research/editorial-policies/reporting-standards#availability-of-data> |  |
| All published manuscripts reporting original research in Nature Portfolio journals must include a data availability statement, as a separate section before the References and under the heading 'Data Availability'.   The data availability statement must make the conditions of access to the “minimum dataset” that are necessary to interpret, verify and extend the research in the article, transparent to readers.   This minimum dataset may be provided through deposition in public community/discipline-specific repositories, custom proprietary repositories or general repositories like Figshare, Zenodo and Dryad. Providing large datasets in supplementary information is strongly discouraged and the preferred approach is to make data available in repositories. Scientific Data, a Nature Portfolio journal, maintains a list of approved and recommended data repositories to support researchers seeking suitable repositories for their data (https://www.nature.com/sdata/policies/repositories).  The Data Availability Statement should also reference any source data published alongside the paper.  If DOIs are provided, we also strongly encourage including these in the Reference list (authors, title, publisher (repository name), identifier, year).  For clinical datasets or third party data, please ensure that the statement adheres to our policy (https://www.nature.com/nature-research/editorial-policies/reporting-standards#availability-of-data) | We uploaded all the data that generated the figures in our paper to Zenodo and this information is included in the ‘Data Availability’ statement:  The data that support the plots within this manuscript and other findings of this study are available on Zenodo (https://doi.org/10.5281/zenodo.5565401). |
| Please use the following template to provide all the information stated above:  The XX data generated in this study have been deposited in the YY database under accession code ZZ [add hyperlink here]. The XX data are available under restricted access for {insert reason}, access can be obtained by {explain how}. The raw XX data are protected and are not available due to data privacy laws. The processed XX data are available at YY. The XX data generated in this study are provided in the Supplementary Information/Source Data file. The XX data used in this study are available in the YY database under accession code ZZ [Add hyperlink here]. | We uploaded all the data that generated the figures in our paper to Zenodo and this information is included in the ‘Data Availability’ statement:  The data that support the plots within this manuscript and other findings of this study are available on Zenodo (https://doi.org/10.5281/zenodo.5565401). |
| Specific advice on your Data Availability Statement: |  |
| In the Data Availability Statement you state "All data generated or analysed during this study are available within the paper and its Supplementary Information" but the submission do not have any supplementary information. please consider changing this statement or uploading a supplementary file. Also please consider making this data available in a publicly accessible repository, or explain why the data can only be made available from the authors on request. | We have revised our ‘Data Availability’ statement:  The data that support the plots within this manuscript and other findings of this study are available on Zenodo (https://doi.org/10.5281/zenodo.5565401). |
| An updated lasing reporting summary must be completed and uploaded as a supplementary information file with the revised manuscript. All points on the reporting summary must be addressed; if needed, please revise your manuscript in response to these points. This checklist is published alongside your manuscript online. Please note that this form is a dynamic "smart pdf" and must therefore be downloaded and completed in Adobe Reader, instead of opening it in a web browser. https://www.nature.com/authors/policies/Lasers.pdf | We completed the lasing reporting summary and would upload as a supplementary information file with the revised manuscript. |
|  |  |
| Methods |  |
| Our guidance: | Your response: |
| Sufficient details of the experiments must be provided in the Methods section such that they could be reproduced without reference to published papers. Use of the term "as described previously" is not encouraged. | We have three subsections “Device fabrication”, “Laser self-injection locking” and “Laser noise measurement” which covers sufficient details of the experiments. |
|  |  |
| References |  |
| Our guidance: | Your response: |
| All references must be cited in numerical order. The reference list will be formatted according to the Nature style by our journal production team, however please ensure that references contain all of the information required, eg:  Kurumada, S., Takamori, S. & Yamashita, M. An alkyl-substituted aluminium anion with strong basicity and nucleophilicity. <i>Nat. Chem.</i> <b>12</b>, 36–39 (2020). | We confirmed our paper follows the guide. |
|  |  |
| End matter |  |
| Our guidance: | Your response: |
| Please supply an "Author Contributions" section after the "Acknowledgements" section that refers to all authors. For more information on the Author Contributions statement, please refer to our authorship policy(https://www.nature.com/nature-research/editorial-policies/authorship), and to the following Nature Editorial: https://www.nature.com/articles/4581078a. | We added the "Author Contributions" section and confirmed our paper follows the guide. |
| Please thoroughly review our policy on Competing Interests (http://www.nature.com/authors/policies/competing.html) and include a detailed statement in your final manuscript file, and in our manuscript tracking system. Please ensure the statements are identical in both. Be specific about how each point stated relates to the research, list applicable author initials, and/or patent numbers. If there are no competing interests, a negative statement (“The authors declare no competing interests”) must be included. | We added the "Competing Interests" section and confirmed our paper follows the guide. |
| Nature Portfolio defines Competing Interest (CI) as financial and non-financial interests (including but not limited to funding, employment, stocks, shares, patents, personal or professional relationships with individuals or institutions, and unpaid membership advocacy) that could be perceived to directly undermine the objectivity, integrity, and value of a publication, or could be seen as having an influence on the judgments and actions of authors with regard to objective data presentation, analysis, and interpretation.  Please thoroughly review our policy on Competing Interests and include a detailed statement both in your final manuscript file and in our manuscript tracking system. Please ensure the statements are identical in both. Be specific about how each point stated relates to the research and list applicable author initials, and/or patent numbers.  If there are no competing interests, a negative statement must be included. | We added the “Competing Interests” section:  JEB is a cofounder of Quintessent and Nexus Photonics, whose focus is in related fields. |
| <https://www.nature.com/nature-research/editorial-policies/competing-interests> |  |
| Please confirm that all relevant funding awarded to each author is described in the Acknowledgements section. List each grant number, followed by the initials of the author who received it. | We wrote in the "Acknowledgements" section:  We acknowledge support from the Defense Advanced Research Projects Agency (DARPA) STTR project (W911NF-19-C-0003). |
|  |  |
| Preparing your manuscript files |  |
| Our guidance: | Your response: |
| Unless otherwise stated please limit individual file sizes to approximately 30MB. We strongly encourage the use of repositories for large datasets or source data due to size considerations. | Our total file size is around 3MB and follows the file size limit. |
| Please supply the main manuscript file in either Microsoft Word or LaTeX format | We supply the main manuscript file in Microsoft Word. |
| Please provide figures as individual vector files with editable text. Acceptable file types for figures are .ai, .eps, .pdf or ChemDraw for fully editable vector-based art. For detailed guidance on figure preparation, see https://www.nature.com/documents/aj-artworkguidelines.pdf | Our figures are provided as .pdf vector files. |
| The use or adaptation of previously published images is strongly discouraged. If this is unavoidable, please request the necessary rights documentation to re-use such material from the relevant copyright holders and return this to us when you submit your revised manuscript. Please check whether your manuscript or Supplementary Information contain third-party images, such as figures from the literature, stock photos, clip art or commercial satellite and map data.  For more information on what constitutes ownership by a third party, please contact our Editorial Assistant at naturecommunications@nature.com | No previously published images are used in our paper. |
|  |  |
|  |  |
| Forms to complete |  |
| Our guidance: | Your response: |
| **Editorial Policy Checklist** |  |
| Please update and upload a final version of the Editorial Policy Checklist with your revised manuscript files. A blank Editorial Policy Checklist can be found via the link below. Note that this form is a dynamic ‘smart pdf’ and must be downloaded and completed in Adobe Reader. |  |
| Please update your current checklist or download from: |  |
| <https://www.nature.com/documents/nr-editorial-policy-checklist.zip> | We completed the Editorial Policy Checklist and would upload with the revised manuscript files. |
|  |  |
|  |  |
| **You will need to upload:** |  |
| Editorial Policy Checklist | Confirmed |
| Completed Third Party Rights Table (if relevant) |  |
| A completed copy of this checklist | Confirmed |
| The main article file in Microsoft Word format - please supply a version with tracked changes and a version with tracked changes accepted | Confirmed |
| Separate Figure files | Confirmed |
| Inventory of Supporting Information |  |
| A Supplementary Information file |  |
| Lasing Reporting Summary | Confirmed |
|  |  |
